# Weighted *p*-norm distance t kernel SVM classification algorithm based on improved polarization

**DOI:** 10.1038/s41598-022-09766-w

**Published:** 2022-04-13

**Authors:** Wenbo Liu, Shengnan Liang, Xiwen Qin

**Affiliations:** 1 School of Mathematics and Statistics, Qiannan Normal University for Nationalities, Duyun, 558000 Guizhou China; 2 Key Laboratory of Complex Systems and Intelligent Optimization of Qiannan, Duyun, 558000 Guizhou China; 3 School of Mathematics and Statistics, Changchun University of Technology, Changchun, 130012 Jilin China

**Keywords:** Computer science, Statistics, Scientific data

## Abstract

The kernel function in SVM enables linear segmentation in a feature space for a large number of linear inseparable data. The kernel function that is selected directly affects the classification performance of SVM. To improve the applicability and classification prediction effect of SVM in different areas, in this paper, we propose a weighted *p*-norm distance t kernel SVM classification algorithm based on improved polarization. A t-class kernel function is constructed according to the t distribution probability density function, and its theoretical proof is presented. To find a suitable mapping space, the t-class kernel function is extended to the *p*-norm distance kernel. The training samples are obtained by stratified sampling, and the affinity matrix is redefined. The improved local kernel polarization is established to obtain the optimal kernel weights and kernel parameters so that different kernel functions are weighted combinations. The cumulative optimal performance rate is constructed to evaluate the overall classification performance of different kernel SVM algorithms, and the significant effects of different *p*-norms on the classification performance of SVM are verified by 10 times fivefold cross-validation statistical comparison tests. In most cases, the results using 6 real datasets show that compared with the traditional kernel function, the proposed weighted *p*-norm distance t kernel can improve the classification prediction performance of SVM.

## Introduction

In the 1990s, Vapnik systematically introduced statistical learning theory and proposed the SVM algorithm^1^. Due to its excellent performance in the field of text mining^2^ and fault diagnosis^3^, SVM gradually became the mainstream technology of machine learning methods and directly promoted the climax of statistical learning development. The study of the kernel method was officially initiated based on the great success of SVM, and SVM promoted the rapid popularization and application of the kernel method. The kernel method has gradually expanded into many fields of machine learning, such as pattern recognition^4^, feature selection^5^, and deep learning^6,7^. The kernel function directly determines the performance of the SVM classification algorithm^8^ and various kernel methods because a proper kernel function can map samples to an appropriate feature space. In an appropriate feature space, similar samples are close together and different samples are far apart. A kernel function is introduced to greatly improve the accuracy, recognition rate, and dimension reduction efficiency of machine learning algorithms.

Subsequently, many methods based on the kernel technique have been proposed. Schkopf^9^ et al. proposed a kernel trick so that principal component analysis could be utilized as a nonlinear dimension reduction technique. As a result, nonlinear mapping from high-dimensional space to low-dimensional space can be achieved and the performance of the learner is improved. Mika^10^ introduced the kernel function into linear discriminant analysis (LDA), which is also known as KLDA. KLDA can address the nonlinear data analysis problem and can achieve higher accuracy than LDA. Si proposed a new and improved kernel partial least squares method to address nonlinear characteristics in industrial processes^11^. Some kernel functions have been proposed for specific fields. For example, Huma^12^ et al. proposed the application of a string kernel in natural language processing to improve the efficiency of text classification. Bernhard^13^ et al. studied the application of kernel methods in the field of bioinformatics.

The above methods are only based on a single kernel. Because different kernel functions have different characteristics, the performance of kernel functions varies greatly in different application scenarios. When the sample size is large, the multidimensional data are irregular or the data are not evenly distributed in the feature space. Therefore, it is not reasonable to map the training set directly by a single kernel^14,15^. To improve the flexibility and applicability of the kernel function, multiple kernel functions are combined, i.e., multiple kernel learning. Multiple kernel learning has been a long-standing, well-known and practical research direction in machine learning. Gone^16^ provided a taxonomy and review of several multiple kernel learning (MKL) algorithms. They concluded that multiple kernel learning is useful in practice and that a better MKL algorithm could be devised for improved accuracy and decreased complexity and training time. In recent years, many multiple kernel methods have been proposed to solve specific problems. Rakotomamonjy^17^ proposed a simple MKL algorithm. In the weighted 2-norm regularization form, an additional 1-norm constraint is applied to the multikernel weight coefficients, which provides a new idea for multiple kernel learning based on mixed norm regularization. Fan^18^ proposed a multiple random empirical kernel learning machine (MREKLM), which adopts the random projection idea to map samples into multiple low-dimensional empirical feature spaces with lower computational complexity. Li^19^ proposed the multiple kernel learning support vector machine particle swarm optimization model to identify pulmonary nodules and obtained better recognition efficiency. Gao^20^ proposed a multiple kernel learning method with the Mahalanobis distance to classify hyperspectral images. Based on the linear weighted combination of the Mahalanobis basic kernel, the hyperspectral data are mapped to a feature space with a smaller intraclass distance and larger interclass distance, and then they are classified to improve the prediction accuracy. Wang^21^ proposed a new model parameter selection method for support vector machines based on adaptive fusion of multiple kernel functions and realized adaptive selection of the multiple kernel function weighted coefficient, kernel parameters and regression parameters. Ergul^22^ proposed a multiple composite kernel extreme learning machine for hyperspectral images, and the obtained results were presented comparatively along with state-of-the-art standard machine learning.

The multiple kernel model has better applicability and flexibility than the single kernel model. The above works have proven that the interpretability of the decision function can be enhanced and the performance of the learner can be boosted by using multiple kernels instead of a single kernel. In the multiple kernel framework, the convex combination of several single kernels,$\sum\nolimits_{i = 1}^{M} {\omega_{i} \kappa_{i} } ,\sum\nolimits_{i = 1}^{M} {\omega_{i} } = 1$ is the most common form. The key to multiple kernel learning is the selection of a basic kernel and the calculation of weight coefficients. We can use the existing kernel as the basic kernel or create a new kernel according to kernel construction theory to use as the basic kernel^23^. There are two main ways to calculate the weight coefficients: heuristic algorithms^24^ and optimization models. The former needs to be associated with the performance of subsequent classifiers, so it is too time-consuming, while the latter has strict theory and lower computational complexity. Examples of typical optimization models are described as follows. Lanckriet^25^ obtained the weighted kernel matrix from data based on a semidefinite programming idea and solved the optimal weight coefficient. Sonnenburg^26^ rewrote the convex quadratic constrained quadratic programming in reference^25^ into a semi-infinite linear programming problem to solve the kernel weight. The gradient descent method was always adopted to optimize the weight by some researchers^27,28^.

Obviously, the multiple kernel model consists of several basic single kernels. The expression of the single kernel function often determines the multiple kernel performance. Single kernel functions have the advantage of simple expression and fewer parameters over multiple kernel functions and can solve specific domain problems. Their deficiency lies in the fixed expression form, which results in poor universality. To solve this problem, a more flexible multiscale kernel was introduced^29,30^. In addition, according to distance metric learning theory, samples are mapped from the original space to the feature space so that the performance obtained in the feature space is better than that in the original space^31^. Obtaining a suitable space is essentially determining the proper distance metric. Therefore, the t class kernel function with multiscale form is constructed. To obtain a suitable distance metric, the t kernel is generalized to the *p*-norm t kernel.

In this study, a weighted *p*-norm distance t kernel (W*p*NDtK) SVM classification algorithm based on improved polarization is proposed for solving basic kernel construction and weight coefficient computation in a multiple kernel model. The main contribution of this paper is as follows. We construct a t-class kernel and provide a theoretical proof. To map the sample to a more suitable feature space, we generalize the t-class kernel as a weighted *p*-norm t-class kernel and give its properties. We define the affinity matrix and build an objective function of weight coefficients and kernel parameters according to local kernel polarization. The objective function is solved by the local gradient and the generalized Lagrange multiplier algorithm. The cumulative optimal performance rate is constructed to measure the overall classification performance of SVM algorithms with different kernels. The significance of the *p*-norm distance on SVM classification performance is verified based on the paired data t test with 10 times fivefold cross-validation. Through a large number of experiments on 6 real datasets, the results show that SVM classification prediction can appropriately improve performance when using W*p*NDtK compared with the traditional kernel function.

This paper is organized as follows. In "Introduction" section, we introduce the development and application of the kernel method and the optimal solution of weight coefficients in multiple kernel learning. The basic SVM model with multiple kernels is introduced in "Kernel support vector machine" section. In "t Class kernel and its generalization: section, we describe the construction of a weighted *p*-norm distance t kernel and provide a theoretical proof. In "Establishment and solution of the multiple kernel model" section, we describe the construction of the optimal model of weight coefficients and kernel parameters. The flow of the weighted *p*-norm t kernel SVM classification algorithm is shown in Weighted *p*-norm t kernel SVM classification algorithm" section. Our experimental studies and an evaluation of the performance of the proposed W*p*NDtK SVM algorithm are presented in "Experimental results and analysis" section. The paper is concluded in "Conclusions" section and suggestions for future work are provided.

## Kernel support vector machine

A support vector machine is a classification algorithm for binary classification problems and is based on the theory of structural risk minimization. Of course, SVM can also be extended to multiclass classification learning problems. The basic SVM model is a maximum interval linear classifier defined in the feature space. By introducing the kernel function, SVM essentially becomes a nonlinear classifier. The basic principle of kernel SVM is given as follows.

Given the training dataset $T = \{ ({\mathbf{x}}_{i} ,y_{i} )\left| {x_{i} \in R^{d} ,y_{i} \in \{ + 1, - 1\} } \right.,i = 1,2,...,n\}$, where ${\mathbf{x}}_{i}$ is the $d$ dimensional input vector and $y_{i}$ is its class label. SVM can be formalized into the following convex quadratic programming problem.

1 $$ \begin{aligned} & \mathop {\min }\limits_{\omega ,b,\xi } \frac{1}{2}\left\| \omega \right\|^{2} + C\sum\limits_{i = 1}^{n} {\xi_{i} } \\ & s.t.\,y_{i} \left[ {\omega^{T} \phi ({\mathbf{x}}_{i} ) + b} \right] \ge 1 - \xi_{i} ,\xi_{i} \ge 0,i = 1,2, \ldots ,n, \\ \end{aligned} $$

where $\omega$ indicates the normal vector of the classification hyperplane, $C$ is a predefined positive trade-off parameter between model simplicity and classification error, $\xi_{i}$ is the vector of slack variables, $\phi (x)$ is the feature vector mapped from $x$, and $b$ is the bias term of the separating hyperplane. The goal of SVM is to maximize the interval $2/\left\| \omega \right\|$.

The dual formulation of Model (1) is generally used when solving SVM

2 $$ \begin{gathered} \mathop {\,\;\max }\limits_{\alpha } \frac{1}{2}\sum\limits_{i = 1}^{n} {\sum\limits_{j = 1}^{n} {\alpha_{i} \alpha_{j} y_{i} y_{j} } \phi ({\mathbf{x}}_{i} ) \cdot \phi ({\mathbf{x}}_{j} )} - \sum\limits_{i = 1}^{n} {\alpha_{i} } \hfill \\ = \mathop {\max }\limits_{\alpha } \frac{1}{2}\sum\limits_{i = 1}^{n} {\sum\limits_{j = 1}^{n} {\alpha_{i} \alpha_{j} y_{i} y_{j} } \kappa ({\mathbf{x}}_{i} ,{\mathbf{x}}_{j} )} - \sum\limits_{i = 1}^{n} {\alpha_{i} } \hfill \\ s.t.\,\sum\limits_{i = 1}^{n} {\alpha_{i} y_{i} } = 0,0 \le \alpha_{i} \le C,i = 1,2,...,n \hfill \\ \end{gathered} $$

where $\kappa ({\mathbf{x}}_{i} ,{\mathbf{x}}_{j} ) = \phi ({\mathbf{x}}_{i} ) \cdot \phi ({\mathbf{x}}_{j} )$ is the kernel function and $\alpha_{i}$ is the Lagrangian multiplier. The bias term $b$ can be solved by the support vector in the training dataset. Its specific form is as follows:

3 $$ b = \frac{1}{{n_{s} }}\left( {\sum\limits_{s = 1}^{{n_{s} }} {y_{s} } - \sum\limits_{i = 1}^{n} {\alpha_{i} y_{i} \kappa ({\mathbf{x}}_{i} ,{\mathbf{x}}_{s} )} } \right) $$

where ${\mathbf{x}}_{s}$ is the support vector and $n_{s}$ is the number of support vectors.

The final SVM classifier is

4 $$ \begin{gathered} f({\mathbf{x}}) = \omega^{T} \phi ({\mathbf{x}}) + b \hfill \\ \quad \,\;\;\; = \sum\limits_{i = 1}^{n} {\alpha_{i} y_{i} \kappa ({\mathbf{x}},{\mathbf{x}}_{i} )} + b. \hfill \\ \end{gathered} $$

For kernel SVM, the selection of the kernel function is the key to the classification performance of SVM. If the kernel function is not properly selected, the sample is mapped to an inappropriate space, which leads to a poor classification effect. To improve the performance, it is necessary to constantly explore the new kernel functions. Since different kernels are applicable to different areas, the most straightforward idea is to combine several different kernels to integrate the advantages of different kernels.

The simplest and most common way to construct a multiple kernel model is to directly combine some single kernels into convex combinations, and the basic form of this concept is as follows.

5 $$ \begin{gathered} \kappa (x,y) = \omega_{1} \kappa_{1} (x,y) + \omega_{2} \kappa_{2} (x,y) + ... + \omega_{M} \kappa_{M} (x,y) \hfill \\ \;\quad \quad {\kern 1pt} \;{\kern 1pt} {\kern 1pt} = \sum\limits_{i = 1}^{M} {\omega_{i} \kappa_{i} (x,y)} \hfill \\ \end{gathered} $$

where $\kappa_{i} (x,y)$ is the basic kernel function, $\omega_{i}$ is the kernel weight and $\sum\nolimits_{i = 1}^{M} {\omega_{i} } = 1$. We can combine existing kernels or construct new classes of kernels. For the determination of kernel weight, a heuristic algorithm or optimization model can be used to solve the weight. The optimization model is used in this work to solve $\omega_{i}$. Section "The optimization of kernel weight and kernel parameter" provides more details.

According to Model (2), the dual formulation of SVM with multiple kernels is as follows.

6 $$ \begin{gathered} \mathop {\max }\limits_{\alpha } \frac{1}{2}\sum\limits_{i = 1}^{n} {\sum\limits_{j = 1}^{n} {\alpha_{i} \alpha_{j} y_{i} y_{j} } \left( {\sum\limits_{k = 1}^{M} {\omega_{k} \kappa_{k} (x,y)} } \right)} - \sum\limits_{i = 1}^{n} {\alpha_{i} } \hfill \\ s.t.\,\sum\limits_{i = 1}^{n} {\alpha_{i} y_{i} } = 0,0 \le \alpha_{i} \le C,i = 1,2,...,n. \hfill \\ \end{gathered} $$

## t Class kernel and its generalization

In many practical tasks, samples are often linearly indivisible. Therefore, it is necessary to select the appropriate kernel function to map the samples to an appropriate feature space so that the samples are linearly separable in the feature space. If the kernel function is not properly selected, the sample cannot be linearly segmented in the feature space, resulting in poor SVM classification performance. Therefore, kernel functions directly determine the performance of SVM classification. This encourages us to construct new types of kernel functions to adapt to different fields. Inspired by the t distribution probability density function, a t class kernel function is constructed. For this kernel to have better flexibility and applicability, it is extended to the *p*-norm distance t kernel, and a reasonable distance measurement can be obtained by adjusting the norm.

### *p*-norm t kernel

#### **Theorem 1**

[32] *Suppose that* $f:X \to R$ *is a bounded continuous integrable function. Then,* $k(x - x^{\prime}) = f(x - x^{\prime})$ *is a kernel function if and only if* $f(0) > 0$ *and its Fourier transform.*

7 $$ \tilde{f}(\omega ) = \int_{ - \infty }^{ + \infty } {f(x)e^{ - i\omega x} dx} \ge 0. $$

#### **Theorem 2**

*When* $n \to + \infty$, *the t distribution probability density function.*

8 $$ f(t) = \frac{{\Gamma (\frac{n + 1}{2})}}{{\sqrt {n\pi } \Gamma (\frac{n}{2})}}(1 + \frac{{t^{2} }}{n})^{{ - \frac{n + 1}{2}}} $$

*is the kernel function, where* $\Gamma ( \cdot )$ *is the gamma function*.

#### ***Proof***

Let $\left| x \right| = t^{2} ,x \in ( - \infty ,\infty )$, Eq. (5) is transformed into.

$$ f(x) = \frac{{\Gamma (\frac{n + 1}{2})}}{{\sqrt {n\pi } \Gamma (\frac{n}{2})}}(1 + \frac{\left| x \right|}{n})^{{ - \frac{n + 1}{2}}} $$

and $$f(0) = \frac{{\Gamma (\frac{n + 1}{2})}}{{\sqrt {n\pi } \Gamma (\frac{n}{2})}} > 0$$.

$$ \tilde{f}(\omega ) = \mathop {\lim }\limits_{n \to + \infty } \int\limits_{X} {f(x)e^{ - i\omega x} } dx $$

$$ \begin{gathered} = \int\limits_{X} {\mathop {\lim }\limits_{n \to + \infty } f(x)e^{ - i\omega x} } dx \hfill \\ = \int_{ - \infty }^{ + \infty } {\mathop {\lim }\limits_{n \to + \infty } \frac{{\Gamma (\frac{n + 1}{2})}}{{\sqrt {n\pi } \Gamma (\frac{n}{2})}}(1 + \frac{\left| x \right|}{n})^{{ - \frac{n + 1}{2}}} e^{ - i\omega x} dx} \hfill \\ { = }\int_{ - \infty }^{ + \infty } {\frac{1}{{\sqrt {2\pi } }}e^{{ - \frac{\left| x \right|}{2}}} e^{ - i\omega x} dx} \hfill \\ \end{gathered} $$

where $e^{{ - \frac{\left| x \right|}{2}}}$ is the Laplacian kernel function. According to Theorem 1,

$$\int_{ - \infty }^{ + \infty } {e^{{ - \frac{\left| x \right|}{2}}} e^{ - i\omega x} dx} \ge 0,$$

Therefore,

$$ \tilde{f}(\omega ){ = }\int_{ - \infty }^{ + \infty } {\frac{1}{{\sqrt {2\pi } }}e^{{ - \frac{\left| x \right|}{2}}} e^{ - i\omega x} dx} \ge 0 $$

When $n \to \infty$, the function

9 $$ f(x) = \frac{{\Gamma (\frac{n + 1}{2})}}{{\sqrt {n\pi } \Gamma (\frac{n}{2})}}(1 + \frac{\left| x \right|}{n})^{{ - \frac{n + 1}{2}}} $$

is the kernel function.

Theorem 2 shows that when the sample size is sufficiently large, the probability density function of the t distribution can be used as the kernel function. The number of $n$1 that should be taken is often determined by experimental analysis. For the convenience of kernel function application, Corollary 1 is given as follows.

#### **Corollary 1**

*When* $n{ = }1$, Eq. (6) *is equivalent to*

10 $$ f(x) = \frac{1}{\pi (1 + \left| x \right|)}. $$

Then, Eq. (10) is the kernel function.

By generalizing the kernel function in Corollary 1, Corollary 2 is obtained as follows.

#### **Corollary 2**

*Let*

11 $$ f(x) = c\left( {\left. {\frac{1}{1 + \left| x \right|}} \right)} \right.^{v} $$

*where* $c > 0,0 < v \le {1}$*; then,* Eq. (11) *is the kernel function.*

#### *Proof*

When, $0 < \frac{1}{1 + \left| x \right|} \le 1,c > 0,0 < v \le {1}$, we have

$$ \begin{aligned}&c\left( {\left. {\frac{1}{1 + \left| x \right|}} \right)} \right. \le c\left( {\left. {\frac{1}{1 + \left| x \right|}} \right)} \right.^{v}\\ &{0} \le \int_{ - \infty }^{ + \infty } {\frac{c}{1 + \left| x \right|}e^{ - i\omega x} dx} \le \int_{ - \infty }^{ + \infty } {c\left( {\left. {\frac{1}{1 + \left| x \right|}} \right)} \right.^{v} e^{ - i\omega x} dx} \end{aligned}$$

Therefore, $f(x) = c\left( {\left. {\frac{1}{1 + \left| x \right|}} \right)} \right.^{v}$ is the kernel function. □

The kernel parameter in Corollary 2 ranges from 0 to 1. We can consider expanding the range of $v$ to increase the applicability of the kernel function.

#### **Theorem 3**

^33^$X \subset R^{n}$,$f:(0,\infty ) \to R$,$\kappa$ *is the function defined on* $X \times X$ *and* $\kappa ({\mathbf{x}},{\mathbf{z}}) = f(\left\| {{\mathbf{x}} - {\mathbf{z}}} \right\|^{2} )$. *When* $f$ *is completely monotone,* $\kappa ({\mathbf{x}},{\mathbf{z}})$ *is a positive definite kernel.*

#### **Corollary 3**

*When* $$c > 0,v > 0$$,

12 $$ f(x) = c\left( {\left. {\frac{1}{1 + x}} \right)} \right.^{v} $$

*is the kernel function, where* $x > 0$.

#### *Proof*

$$ \begin{aligned} & f^{(n)} (x) = ( - 1)^{n} cv(v + 1)...(v + (n - 1))(1 + x)^{ - (v + n)} \\ & ( - 1)^{n} f^{(n)} (x) = ( - 1)^{2n} cv(v + 1)...(v + (n - 1))(1 + x)^{ - (v + n)} \\ & \;\quad \quad \,\quad \quad \;\, = cv(v + 1)...(v + (n - 1))(1 + x)^{ - (v + n)} \\ \end{aligned} $$

When $c > 0,v > 0$ and $( - 1)^{n} f^{(n)} (x) \ge 0$, $f(x)$ is completely monotone. According to Theorem 3, $f(x)$ is the kernel function. □

According to the complete monotonicity of the function, Corollary 3 expands the range of kernel parameters on the basis of Corollary 2, which provides more choices for us to use the kernel function.

In practical applications, Eq. (12) is in the following form:

13 $$ \kappa ({\mathbf{x}}_{i} ,{\mathbf{x}}_{j} ){ = }\left( {\frac{c}{{1 + \left\| {{\mathbf{x}}_{i} - {\mathbf{x}}_{j} } \right\|_{2} }}} \right)^{v} $$

where the number 2 indicates the 2-norm. To find an appropriate distance measure in the mapped feature space, the Euclidean distance in Eq. (10) is generalized to the *p*-norm distance, and we can obtain

14 $$ \kappa ({\mathbf{x}}_{i} ,{\mathbf{x}}_{j} ){ = }\left( {\frac{c}{{1 + \left\| {{\mathbf{x}}_{i} - {\mathbf{x}}_{j} } \right\|_{p} }}} \right)^{v} $$

where $p$ is the *p*-norm. Equation (14) is called the *p*-norm distance t class kernel for the short *p*-norm t kernel.

### The properties of the kernel function

Since the kernel function constructed in "*p*-norm t kernel" section is eventually extended to the form of Eq. (14), the corresponding properties are given in this section. We also discuss whether this kernel function is reasonable.

#### *Property 1*

When $c > 0,v > 0$, $f(x) = c\left( {\left. {\frac{1}{1 + x}} \right)} \right.^{v}$ is a decreasing function of $x$, and.

$d_{ij} = \left\| {{\mathbf{x}}_{i} - {\mathbf{x}}_{j} } \right\|_{p}$ is the *p*-norm distance of any two samples. According to Property 1, the closer the sample is, the larger the kernel value is, and vice versa. When $x{ = 0}$, the kernel function is at its maximum value. This shows that the kernel function can describe the similarity between samples well. The larger the kernel value is, the higher the similarity between samples.

#### *Property 2*

The function $f(x) = c\left( {\left. {\frac{1}{1 + x}} \right)} \right.^{v}$ has multiscale characteristics, where $c$ and $v$ are the scale parameters.

**Figure 1:**
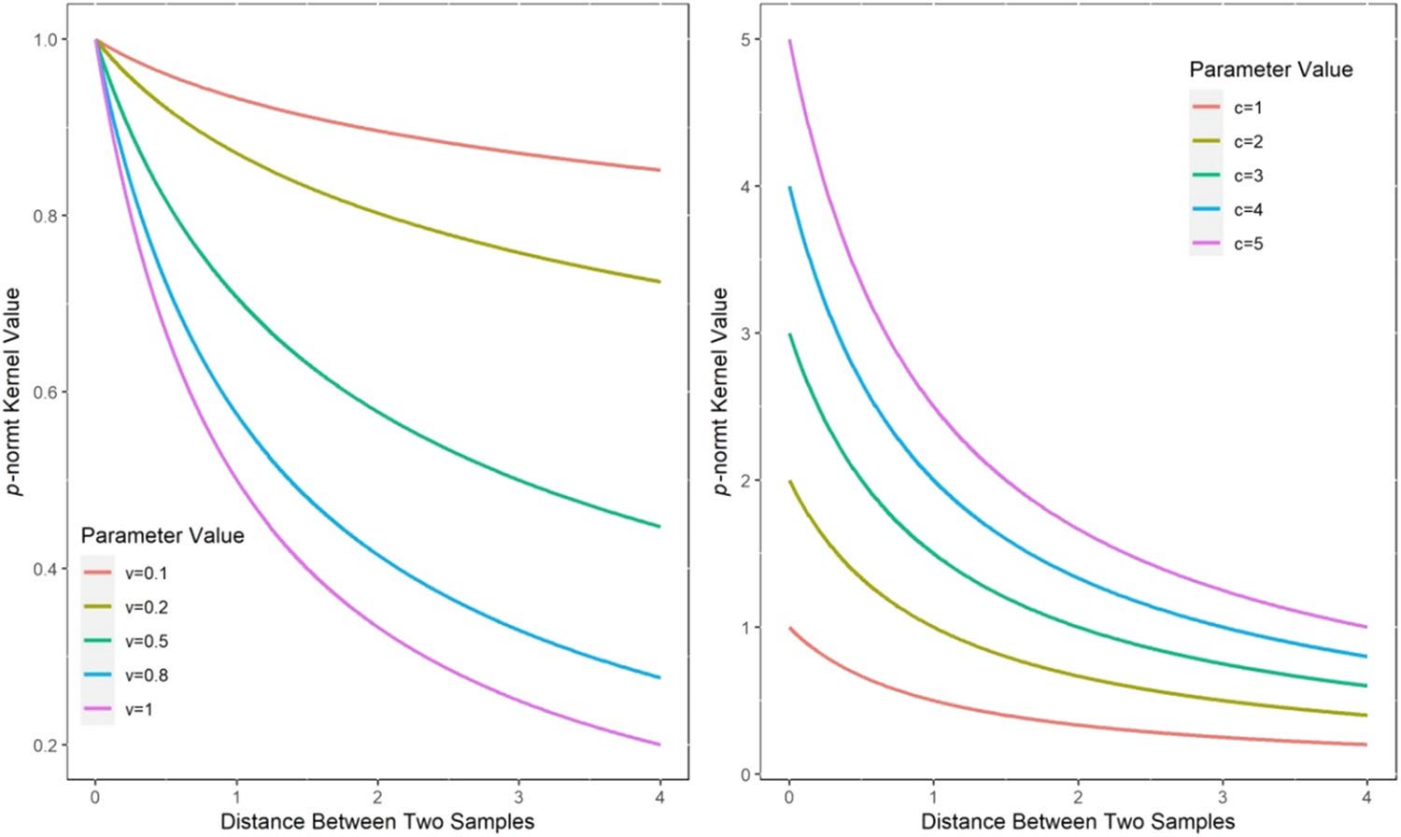
*p*-norm distance t-kernel value under different scale parameters.

Property 2 is illustrated by function graphs, which are drawn by fixing $c{ = 1}$ and $v = 1$, as shown in Fig. 1.

When the scale parameters $c$ and $v$ are small, the kernel function can adapt to the samples with drastic changes, and when the scale parameters are large, the kernel function can adapt to the samples with gentle changes^34^ so that it has better adaptability in processing complex data. Similar to the Gaussian kernel function, the constructed kernel function in Eq. (14) is also a typical multiscale kernel.

## Establishment and solution of the multiple kernel model

### Weighted kernel function

Because different kernel functions have different characteristics, their performance will be significantly different for different types of datasets. To make the kernel function more flexible in application, the multiple kernel learning model is formed by kernel combination. Using multiple kernels instead of a single kernel can enhance the interpretability of the decision function and result in better performance than a single kernel^35^.

When the *p*-norm t kernel constructed in "*p*-norm t kernel" section is combined, we can obtain the combination kernel as follows.

15 $$ \sum\limits_{s = 1}^{M} {\omega_{s} \left( {\frac{c}{{1 + \left\| {{\mathbf{x}}_{i} - {\mathbf{x}}_{j} } \right\|_{p} }}} \right)^{{v_{s} }} } $$

Under the framework of a multiple kernel learning model, the representation of original samples in feature space is transformed into basic kernel selection and the calculation of weight coefficients. Each basic kernel corresponds to a basic feature space and how to fuse these basic feature spaces to obtain a suitable combined feature space. That is, the data can be better represented in the combined feature space to improve the classification prediction performance. Obtaining the combined feature space is essentially a problem of optimal calculation of weight coefficients.

Currently, there are two main methods to calculate the weight coefficient: a heuristic algorithm and an optimization algorithm. In this work, an optimization method that has a more rigorous theory is adopted to solve the weight coefficients. The key step to establish the optimization model is to give the objective function. In this study, the objective function is established based on kernel target alignment, and the optimal solution should maximize the target value. Kernel target alignment only relies on training samples and is unrelated to subsequent classifiers, so the implementation of this strategy is simple and has attracted a large amount of attention. Since the kernel function contains hyperparameters, the value of the kernel parameters also has a significant impact on the performance of the classification prediction results. Therefore, how to select the appropriate hyperparameters is also a key consideration. A direct approach is to put the kernel parameters and the weight together into the objective function for optimization.

### Kernel target alignment

Kernel target alignment is a parameter optimization criterion established based on matrix alignment. This type of method only relies on training samples and is unrelated to the learning performance of subsequent classifiers. Therefore, the algorithm is simple and quick to implement, and its basic principle is as follows.

Given the training dataset $D = \{ {\mathbf{x}}_{1} ,{\mathbf{x}}_{2} , \ldots ,{\mathbf{x}}_{n} \}$ and class label ${\mathbf{y}} = \{ y_{1} ,y_{2} ,...,y_{n} \}$, $y_{i} \in \{ 1,2,...,k\}$ shows that the dataset has $k$ classes, and ${\rm K} = (\kappa ({\mathbf{x}}_{i} ,{\mathbf{x}}_{j} ))_{n \times n}$ is the kernel matrix. Then, $Y = {\mathbf{yy}}^{T} = (y_{ij} )_{n \times n}$ is the class label matrix and is also called the ideal kernel matrix, where.

$$y_{ij} = \left\{ \begin{gathered} 1,\;\;\,\,y_{i} = y_{j} \hfill \\ - 1,y_{i} \ne y_{j} \hfill \\ \end{gathered} \right.$$.

The goal of the kernel target alignment is to maximize the cosine value between the kernel matrix and the ideal kernel matrix, and its expression is as follows.

16 $$ A({\rm K},Y) = \frac{{ < {\rm K},Y >_{F} }}{{\left\| {\rm K} \right\|_{F} \left\| Y \right\|_{F} }} $$

where $< \cdot , \cdot >_{F}$ is the Frobenius inner product and $\left\| {\, \cdot \,} \right\|_{F}$ is the Frobenius norm. Reference^36^ proves the reliability and practicability of the kernel target alignment and the boundedness of the generalization error of the kernel classifier. On the basis of Eq. (15), Baram proposed kernel polarization inspired by physics^37^. It is defined as the Frobenius inner product.

17 $$ P({\rm K}) = \sum\limits_{i = 1}^{n} {\sum\limits_{j = 1}^{n} {y_{ij} } } {\rm K}({\mathbf{x}}_{i} ,{\mathbf{x}}_{j} ). $$

where $P({\rm K})$ only takes between-class separability into account but neglects the preservation of within-class local structures; therefore, Wang proposed local kernel polarization (LKP)^38^, which is defined as.

18 $$ L({\rm K}) = \sum\limits_{i = 1}^{n} {\sum\limits_{j = 1}^{n} {A_{ij} y_{ij} } } {\rm K}({\mathbf{x}}_{i} ,{\mathbf{x}}_{j} ). $$

The affinity coefficient is defined as

19 $$ A_{ij} = \left\{ \begin{gathered} \exp ( - t\left\| {{\mathbf{x}}_{i} - {\mathbf{x}}_{j} } \right\|_{2} ),y_{i} = y_{j} \hfill \\ 1,\quad \quad \quad \quad \,\,\,{\kern 1pt} \,\;\quad \,\,{\kern 1pt} y_{i} \ne y_{j} \hfill \\ \end{gathered} \right. $$

where $t > 0$ is the adjusting parameter. From Eq. (19), the affinity coefficient $A_{ij}$ is defined by the Gaussian kernel function. Certainly, there should be some other more appropriate manners of defining the affinity coefficient. Therefore, we redefine the affinity coefficient in "The optimization of kernel weight and kernel parameter" section to obtain better results.

### The optimization of kernel weight and kernel parameter

Based on the basic idea of the LKP, an improved local kernel polarization model is constructed to obtain the optimal kernel weights and kernel parameters. The improved part is reflected in the redefinition of the affinity coefficient in the LKP. The specific optimization model is as follows.

20 $$ \begin{gathered} \mathop {\max }\limits_{{\omega ,v_{s} }} \sum\limits_{j = 1}^{n} {\sum\limits_{i = 1}^{n} {A_{ij} y_{ij} \sum\limits_{s = 1}^{M} {\omega_{s} \left( {\frac{c}{{1 + \left\| {{\mathbf{x}}_{i} - {\mathbf{x}}_{j} } \right\|_{{p_{s} }} }}} \right)^{{v_{s} }} } } } \hfill \\ s.t.{\kern 1pt} \;0 \le \omega_{s} \le 1,\,v_{s} > 0,\sum\limits_{s = 1}^{M} {\omega_{s} } = 1. \hfill \\ \end{gathered} $$

By redefining the affinity coefficient, we obtain.

21 $$ A_{ij} = \left\{ \begin{gathered} \frac{1}{{1 + \left\| {{\mathbf{x}}_{i} - {\mathbf{x}}_{j} } \right\|_{p} }},y_{i} = y_{j} \hfill \\ 1,\quad \quad \quad \quad \,\,\,{\kern 1pt} \,,y_{i} \ne y_{j} \hfill \\ \end{gathered} \right. $$

For Model (20), an optimization algorithm combining the local gradient and generalized Lagrange multiplier is adopted^39^. The gradient form of the model is as follows:

$$ \begin{aligned} & \frac{{\partial \sum\limits_{j = 1}^{n} {\sum\limits_{i = 1}^{n} {A_{ij} y_{i} y_{j} \sum\limits_{s = 1}^{M} {\omega_{s} \left( {\frac{c}{{1 + \left\| {{\mathbf{x}}_{i} - {\mathbf{x}}_{j} } \right\|_{{p_{s} }} }}} \right)^{{v_{s} }} } } } }}{{\partial \omega_{s} }} \\ & = \sum\limits_{j = 1}^{n} {\sum\limits_{i = 1}^{n} {A_{ij} y_{i} y_{j} \left( {\frac{c}{{1 + \left\| {{\mathbf{x}}_{i} - {\mathbf{x}}_{j} } \right\|_{{p_{s} }} }}} \right)^{{v_{s} }} } } , \\ & { = }\frac{{\partial \sum\limits_{j = 1}^{n} {\sum\limits_{i = 1}^{n} {A_{ij} y_{i} y_{j} \sum\limits_{s = 1}^{M} {\omega_{s} \left( {\frac{c}{{1 + \left\| {{\mathbf{x}}_{i} - {\mathbf{x}}_{j} } \right\|_{{p_{s} }} }}} \right)^{{v_{s} }} } } } }}{{\partial v_{s} }} \\ & = \sum\limits_{j = 1}^{n} {\sum\limits_{i = 1}^{n} {A_{ij} y_{i} y_{j} \omega_{s} \left( {\frac{c}{{1 + \left\| {{\mathbf{x}}_{i} - {\mathbf{x}}_{j} } \right\|_{{p_{s} }} }}} \right)^{{v_{s} }} } } \log \frac{1}{{1 + \left\| {{\mathbf{x}}_{i} - {\mathbf{x}}_{j} } \right\|_{{p_{s} }} }},s = 1,2,...,M \\ \end{aligned} $$

To facilitate calculation, the parameters in Eq. (20) can be specified in advance, and for convenience $c = 1$.

Equation (20) only contains the weighted *p*-norm t kernel. However, according to different field applications, the *p*-norm t kernel can also be combined with other types of kernel functions to obtain better classification performance.

## Weighted *p*-norm t kernel SVM classification algorithm

According to the construction principle of the *p*-norm t-kernel and the establishment and solving process of the multiple kernel model, the basic flow of the weighted *p*-norm t kernel SVM classification algorithm is as follows.

Input: $Train = \{ ({\mathbf{x}}_{i} ,y_{i} )\left| {{\mathbf{x}}_{i} \in R^{p} } \right.,y_{i} \in Y,i = 1,2,...,n\}$, where $Y = \{ 1,2,...,l\}$ is the class label.

Output: The predicted class $\hat{y}_{i}$ of $Test = \{ {\mathbf{x^{\prime}}}_{i} \left| {{\mathbf{x^{\prime}}} \in R^{p} } \right.\}$, $i = 1,2,...,n^{\prime}$.

Step 1: The dataset is divided into a training set and a test set by k-fold cross stratified sampling.

Step 2: A specific kernel function is selected according to Eq. (5).

Step 3: The affinity coefficient matrix is built according to Eq. (21).

Step 4: According to Eq. (18), the objective function of kernel weight and kernel parameter is established.

Step 5: Based on the training set, the local gradient and generalized Lagrange multiplier^39^ are used to solve Model (20) and obtain the optimal weight coefficients $\omega_{i}$ and kernel parameters $v,\gamma ,d$.

Step 6: The optimal parameters obtained in Step 5 are substituted into Eq. (5).

Step 7: Eq. (5), which is obtained in Step 6, is substituted into Model (6) to obtain the specific dual formulation of the multiple kernel SVM.

Step 8: The training set $Train$ obtained by stratified sampling is used to fit Model (6).

Step 9: The test set is put into the fitted Model (6) to obtain the predicted class label $\hat{y}_{i}$.

In Step 1, stratified sampling is used to prevent class imbalance in the training set and prevent the fitted SVM classification model from having class tendency. The specific form of each single kernel function must be specified in Step 2. In this study, the *p*-norm t-kernel constructed in "*p*-norm t kernel" section is mainly used for weighted combination. According to the experimental analysis in Step 6, to make use of the unique advantages of different kernel functions, the *p*-norm t kernel can also be combined with traditional kernel functions, including the Gaussian kernel and polynomial kernel. Steps 3 to 5 belong to the optimization process of model parameters, including the solution of weight coefficients and kernel parameters. The objective function is established according to the local kernel polarization, and the local gradient and the generalized Lagrange multiplier are used to solve it. Of course, other optimization algorithms can also be adopted. For details, please refer to reference^40^. Steps 6 to 8 fit the multiple kernel SVM model, and Step 9 predicts the test samples based on the fitted model. Finally, a specific evaluation index is used to evaluate the weighted *p*-norm t kernel SVM classification algorithm.

## Experimental results and analysis

### Experimental setting

The experimental environment uses a Windows 10 64-bit operating system with an Intel i7-9700 @ 3.0 GHz CUP and 16 GB memory. The algorithm and experiment proposed in this paper are implemented based on R language (R 3.6.3) coding. Experimental data are from the Broad Institute Genome Data Analysis Center and UCI machine learning library. The specific information is shown in Table 1.

**Table 1:** Data information.

Dataset name	Sample size	Feature	Categories	Data source
Kidney	400	24	2	UCI
Dermatology	366	34	6	UCI
Sonar	208	60	2	UCI
Pima	768	8	2	UCI
Postcode	7291	256	10	^41^
Breast	98	1213	3	BIGDAC yyy^42,43^

UCI: http://archive.ics.uci.edu/ml/index.php.

BIGDAC: http://portals.broadinstitute.org/cgi-bin/cancer/datasets.cgi.

.

We compare the performance of the W*p*Nt + SVM algorithm with the following methods:

(i) Poly + SVM: The polynomial kernel is used in SVM.
(ii) Sig + SVM: The sigmoid kernel is used in SVM.
(iii) Gau + SVM: The Gaussian kernel is used in SVM.
(iv) Lap + SVM: The Laplace kernel is used in SVM.
(v) Simple MKL: The linear combination of kernel approach is used. Two kernel functions are mixed in the experiment, including two Gaussian kernels, one Gaussian kernel and one linear kernel.

To compare the effects of different kernel functions on the performance of the SVM classification algorithm, the experiment used fivefold cross-validation to divide the training set and test set, and the evaluation criteria were classification accuracy, recall, Kappa coefficient^44^ and training time. The training time of the algorithm is related to the range of parameter settings, as it often takes more time to obtain results with good performance. Different from the previous three evaluation indices, the training time of the algorithm is discussed separately in "Comparison experiment" section. Due to the large sample size of the postcode dataset, 10% random sampling is carried out in the training phase to reduce the time. Because of the high dimensionality of the breast dataset, PCA is used to reduce its dimensionality in advance. To evaluate the overall performance level of the W*p*Nt + SVM algorithm, the optimal performance rate is constructed as follows.

22 $$ OPR = \frac{PN}{{MN \times DN \times EN}} $$

where $MN$ is the number of algorithms, $DN$ is the number of datasets, $EN$ is the number of evaluation indices, and $PN$ is the number of W*p*Nt + SVM that reaches the maximum under each evaluation index.

Equation (22) is generalized to obtain the cumulative optimal performance rate (COPR). Its definition is as follows:

23 $$ COPR = \frac{{\sum\limits_{i = 1}^{m} {PN_{i} } }}{MN \times DN \times EN} $$

where $PN_{i}$ is the number of algorithms reaching the $i$ ^th^ maximum under each evaluation index, and $m$ is the number of methods.

### Comparison experiment

For different datasets, SVM classification based on different kernel functions yields different prediction effects. In experimental analysis, to obtain better classification and prediction performance, flexibility is required when encountering different datasets; that is, multiple *p*-norm distance t kernels should be combined or *p*-norm distance t kernels should be combined with traditional kernel functions when encountering different datasets. To reduce the complexity of the experiment, only two kernel functions are combined, and a positive trade-off parameter $C = 1$ is allowed in all the SVM models. After many comparative experiments, different weighted kernel functions are selected for different datasets. The form of weighted kernel functions is mainly as follows.

24 $$ \omega_{1} \left( {\frac{1}{{1 + \left\| {{\mathbf{x}}_{i} - {\mathbf{x}}_{j} } \right\|_{{p_{s} }} }}} \right)^{{v_{1} }} + \omega_{2} \left( {\frac{1}{{1 + \left\| {{\mathbf{x}}_{i} - {\mathbf{x}}_{j} } \right\|_{{p_{s} }} }}} \right)^{{v_{2} }} $$

25 $$ \omega_{1} \left( {\frac{1}{{1 + \left\| {{\mathbf{x}}_{i} - {\mathbf{x}}_{j} } \right\|_{{p_{s} }} }}} \right)^{{v_{1} }} + \omega_{2} \left( {{\mathbf{x}}_{i} \cdot {\mathbf{x}}_{j} } \right)^{d} $$

26 $$ \omega_{1} \left( {\frac{1}{{1 + \left\| {{\mathbf{x}}_{i} - {\mathbf{x}}_{j} } \right\|_{{p_{s} }} }}} \right)^{{v_{1} }} + \omega_{2} \exp \left( {{ - }\gamma \left\| {{\mathbf{x}}_{i} - {\mathbf{x}}_{j} } \right\|_{2} } \right) $$

Equation (24) is applied to the Kidney and Pima datasets, Eq. (25) is applied to the Postcode and Breast datasets, and Eq. (26) is applied to the Dermatology and Sonar datasets. When calculating the kernel weight and the kernel parameters, optimization Model (20) is adopted, and the aforementioned local gradient and generalized Lagrange multiplier method are used to solve the problem. The results are shown in Table 2.

**Table 2:** The optimized result of the weight coefficients and kernel parameters.

Dataset	$\omega_{1}$	$\omega_{2}$	Kernel parameter 1	Kernel parameter 2
Kidney	0.78	0.22	$v_{1} =$ 1.00	$v_{2} =$ 0.80
Dermatology	0.23	0.77	$v_{1} =$ 0.94	$\gamma =$ 0.01
Sonar	0.91	0.29	$v_{1} =$ 0.94	$\gamma =$ 0.04
Pima	0.78	0.22	$v_{1} =$ 0.91	$v_{2} =$ 0.86
Postcode	0.86	0.14	$v_{1} =$ 0.84	$d =$ 1.00
Breast	0.65	0.35	$v_{1} =$ 0.99	$d =$ 1.00

The *p*-norm value is set by the wrapping strategy in Eqs. (24)–(26). The *p*-norm value in $[a,b]$ is set and the step size $\lambda$ is given. For different *p* values, each performance index of $N$ times k-fold cross-validation of the proposed method is calculated, including accuracy, recall and Kappa coefficient. Finally, the *p*-norm value corresponding to the optimal performance index is determined.

For W*p*Nt + SVM algorithm, the objective function with the kernel weight and kernel parameter is established according to the improved local polarization. The local gradient and generalized Lagrange multiplier is adopted to obtain the optimal weights and parameters. For the other comparison algorithms, grid search strategy and k-fold cross validation are used to obtain the optimal parameters.

The different kernel SVM methods are denoted as Poly + SVM, Sig + SVM, Gau + SVM, Lap + SVM, SMKL + SVM and W*p*Nt + SVM. These methods are used to perform fivefold cross-validation classification prediction for the 6 datasets shown in Table 1. The obtained comparative experimental results are shown in Tables 3, 4 and 5, and the optimal results are bolded.

**Table 3:** The fivefold cross-validation classification accuracy based on the SVM algorithm with different kernel functions.

Dataset	Poly + SVM	Sig + SVM	Gau + SVM	Lap + SVM	SMKL + SVM	W*p*Nt + SVM
Kidney	$d = 4$ 0.9575	$\beta = 1\;\theta { = - }8$ 0.9850	$\sigma = 0.1$ **0.9975**	$\sigma = 0.1$ **0.9975**	$\sigma_{1} = 0.01{\kern 1pt} \sigma_{2} = 0.05$ **0.9975**	$p = 1.5$ **0.9975**
Dermatology	$d = 3$ 0.9344	$\beta = 0.1\;\theta { = - 2}$ 0.9672	$\sigma = 0.05$ 0.9645	$\sigma = 0.1$ 0.9699	$\sigma_{1} = 0.01{\kern 1pt} \sigma_{2} = 0.04$ 0.9672	$p = 1.5$ **0.9726**
Sonar	$d = 3$ **0.8559**	$\beta = 0.01\;\theta { = - 1}$ 0.7978	$\sigma = 0.01$ 0.8413	$\sigma = 0.05$ 0.8170	$\sigma_{1} = 0.01{\kern 1pt} \sigma_{2} = 0.02$ 0.8364	$p = 2$ 0.8459
Pima	$d = 2$ 0.7448	$\beta = 1\;\theta { = - 1}$ 0.6771	$\sigma = 0.1$ 0.7643	$\sigma = 0.05$ 0.7735	$\sigma_{1} = 0.01{\kern 1pt} \sigma_{2} = 0.02$ **0.7748**	$p = 2$ 0.7696
Postcode	$d = 2$ 0.9243	$\beta = 0.01\;\theta { = - 2}$ **0.9257**	$\sigma = 0.01$ 0.8432	$\sigma = 0.05$ 0.9230	$\sigma = 0.01,d = 1$ 0.9243	$p = 1.5$ **0.9257**
Breast	$d = 2$ 0.8684	$\beta = 1\;\theta { = - 1}$ 0.7653	$\sigma = 0.01$ 0.8384	$\sigma = 0.05$ 0.6947	$\sigma = 0.02,d = 1$ 0.8684	$p = {2}.5$ **0.8789**

Significant values are in bold.

**Table 4:** The fivefold cross-validation classification recall based on the SVM algorithm with different kernel functions.

Dataset	Poly + SVM	Sig + SVM	Gau + SVM	Lap + SVM	SMKL + SVM	W*p*Nt + SVM
Kidney	$d = 4$ 0.9494	$\beta = 1\;\theta { = - }8$ 0.9875	$\sigma = 0.1$ **0.9979**	$\sigma = 0.1$ **0.9979**	$\sigma_{1} = 0.01{\kern 1pt} \sigma_{2} = 0.05$ **0.9979**	$p = 1.5$ **0.9979**
Dermatology	$d = 3$ 0.9453	$\beta = 0.1\;\theta { = - 2}$ 0.9727	$\sigma = 0.05$ 0.9704	$\sigma = 0.1$ 0.9749	$\sigma_{1} = 0.01{\kern 1pt} \sigma_{2} = 0.04$ 0.9727	$p = 1.5$ **0.9772**
Sonar	$d = 3$ **0.8720**	$\beta = 1\;\theta { = - 1}$ 0.8077	$\sigma = 0.01$ 0.8678	$\sigma = 0.05$ 0.8237	$\sigma_{1} = 0.01{\kern 1pt} \sigma_{2} = 0.02$ 0.8398	$p = 2$ 0.8636
Pima	$d = 2$ 0.7491	$\beta = 1\;\theta { = - 1}$ 0.6820	$\sigma = 0.1$ 0.7761	$\sigma = 0.05$ 0.7883	$\sigma_{1} = 0.01{\kern 1pt} \sigma_{2} = 0.02$ **0.7925**	$p = 2$ 0.7774
Postcode	$d = 2$ 0.9211	$\beta = 0.01\;\theta { = - 2}$ **0.9381**	$\sigma = 0.1$ 0.8693	$\sigma = 0.05$ 0.9358	$\sigma = 0.02,d = 1$ 0.9370	$p = 1.5$ 0.9370
Breast	$d = 2$ 0.8070	$\beta = 1\;\theta { = - 1}$ 0.6377	$\sigma = 0.1$ 0.7820	$\sigma = 0.05$ 0.5789	$\sigma = 0.02,d = 1$ 0.8070	$p = {2}.5$ **0.8158**

Significant values are in bold.

**Table 5:** The fivefold cross-validation classification Kappa coefficient based on the SVM algorithm with different kernel functions.

Dataset	Poly + SVM	Sig + SVM	Gau + SVM	Lap + SVM	SMKL + SVM	W*p*Nt + SVM
Kidney	$d = 4$ 0.9113	$\beta = 1\;\theta { = - }8$ 0.9679	$\sigma = 0.1$ **0.9945**	$\sigma = 0.1$ **0.9945**	$\sigma_{1} = 0.01{\kern 1pt} \sigma_{2} = 0.05$ **0.9945**	$p = 1.5$ **0.9945**
Dermatology	$d = 3$ 0.9174	$\beta = 0.1\;\theta { = - 2}$ 0.9586	$\sigma = 0.05$ 0.9550	$\sigma = 0.1$ 0.9619	$\sigma_{1} = 0.01{\kern 1pt} \sigma_{2} = 0.04$ 0.9585	$p = 1.5$ **0.9654**
Sonar	$d = 3$ **0.7055**	$\beta = 1\;\theta { = - 1}$ 0.5922	$\sigma = 0.01$ 0.6778	$\sigma = 0.05$ 0.6300	$\sigma_{1} = 0.01{\kern 1pt} \sigma_{2} = 0.02$ 0.6692	$p = 2$ 0.6861
Pima	$d = 2$ 0.4154	$\beta = 1\;\theta { = - 1}$ 0.2894	$\sigma = 0.1$ 0.4573	$\sigma = 0.05$ 0.4743	$\sigma_{1} = 0.01{\kern 1pt} \sigma_{2} = 0.02$ **0.4761**	$p = 2$ 0.4741
Postcode	$d = 2$ 0.9148	$\beta = 0.01\;\theta { = - 2}$ **0.9163**	$\sigma = 0.1$ 0.8232	$\sigma = 0.05$ 0.9130	$\sigma = 0.02,d = 1$ 0.9146	$p = 2$ 0.9161
Breast	$d = 2$ 0.7683	$\beta = 1\;\theta { = - 1}$ 0.5935	$\sigma = 0.1$ 0.7106	$\sigma = 0.05$ 0.3879	$\sigma = 0.02,d = 1$ 0.7694	$p = {2}.5$ **0.7862**

Significant values are in bold.

According to the experimental results in Tables 3, 4 and 5, the accuracy of the W*p*Nt + SVM algorithm is optimal for 4 datasets and suboptimal in 1 datasets, the recall of the W*p*Nt + SVM algorithm is optimal for 3 datasets and suboptimal for 1 dataset, and the Kappa coefficient of the W*p*Nt + SVM algorithm is optimal for 3 datasets and suboptimal for 2 datasets. According to Eqs. (18) and (19), the optimal performance rate and cumulative optimal performance rate of W*p*Nt + SVM are calculated as follows.

$$ \begin{aligned} OPR &= \frac{{{4 + }3{ + 3}}}{1 \times 6 \times 3} \approx 0.5566 \\ COPR &= \frac{{(5{ + }3{ + }2) + (1 + 1 + 2)}}{1 \times 6 \times 3} \approx 0.7778 \end{aligned}$$

In the 6 datasets analysed, W*p*Nt + SVM is optimal in 10 cases and suboptimal in 6 cases, and the cumulative optimal performance rate is 0.7778, which is close to 80%. This shows that the *p*-norm t kernel constructed for this study can effectively improve the classification and prediction performance of the SVM algorithm. In addition, the combination of the *p*-norm t kernel with the classical Gaussian kernel and polynomial kernel is often better than the single kernel function. Therefore, multiple learning methods can utilize the advantages of each single kernel effectively.

In classification prediction, the training time of the algorithm is also an important evaluation index. Since the final parameters of the comparison algorithm are determined by the wrapping strategy, the grid search strategy is used to set the range of hyperparameters in advance.

The specific setup information is polynomial kernel:$d = 1:5$, and the step size is 1; Gaussian kernel $\sigma = 0.01:4$, and the step size is 0.01; Laplace kernel:$\sigma = 0.01:1$, and the step size is 0.05; Sigmoid kernel: $\beta = 1:5,\theta = - 10: - 1$, and the step size is 1. The optimization model is used to solve the kernel weights and parameters of the W*p*Nt + SVM algorithm, so there is no need to set parameters in advance. See Table 6 for the specific training time (in minutes) of all algorithms.

**Table 6:** The fivefold cross-validation classification training time based on the SVM algorithm with different kernel functions (minutes).

Dataset	Poly + SVM	Sig + SVM	Gau + SVM	Lap + SVM	SMKL + SVM	W*p*Nt + SVM
Kidney	0.05	0.12	0. 87	0.05	0.64	0.78
Dermatology	0.07	0.18	2.64	0.133	0.54	0.65
Sonar	0.04	0.18	0.60	0.03	0.21	0.29
Pima	4.20	0.29	2.01	0.08	2.53	2.71
Postcode	1.37	3.73	35.25	1.71	8.21	17.53
Breast	0.31	0.36	0.70	0.33	0.32	0.39

According to Table 6, except for the Gau + SVM algorithm, in general, the training time of W*p*Nt + SVM is higher than that of the other comparison algorithms in most cases. It should be emphasized that for Poly + SVM, Sig + SVM, Gau + SVM and Lap + SVM, the training time is dependent on the setting range of the parameters. The optimization model is established to solve the parameters of W*p*Nt + SVM and SMKL + SVM based on the improved local polarization. Therefore, the algorithm proposed in this study does not depend on the setting range of the parameters. The hyperparameter in the Gaussian kernel has the smallest step size compared to other single kernels. The training time of Gau + SVM is significantly higher than that of W*p*Nt + SVM and SMKL + SVM in all datasets except the Pima dataset. This indicates that the training time of Poly + SVM, Sig + SVM, Gau + SVM and Lap + SVM will certainly exceed the training time of W*p*Nt + SVM and SMKL + SVM if the value range of parameters is added and the step size is continuously reduced. When dealing with the large sample data, R or Python's GPU module can be called for training the model. W*p*Nt + SVM can be parallel computing, so that the training time is reduced.

### Statistical measurement comparison test of *p*-norm distance

**Figure 2:**
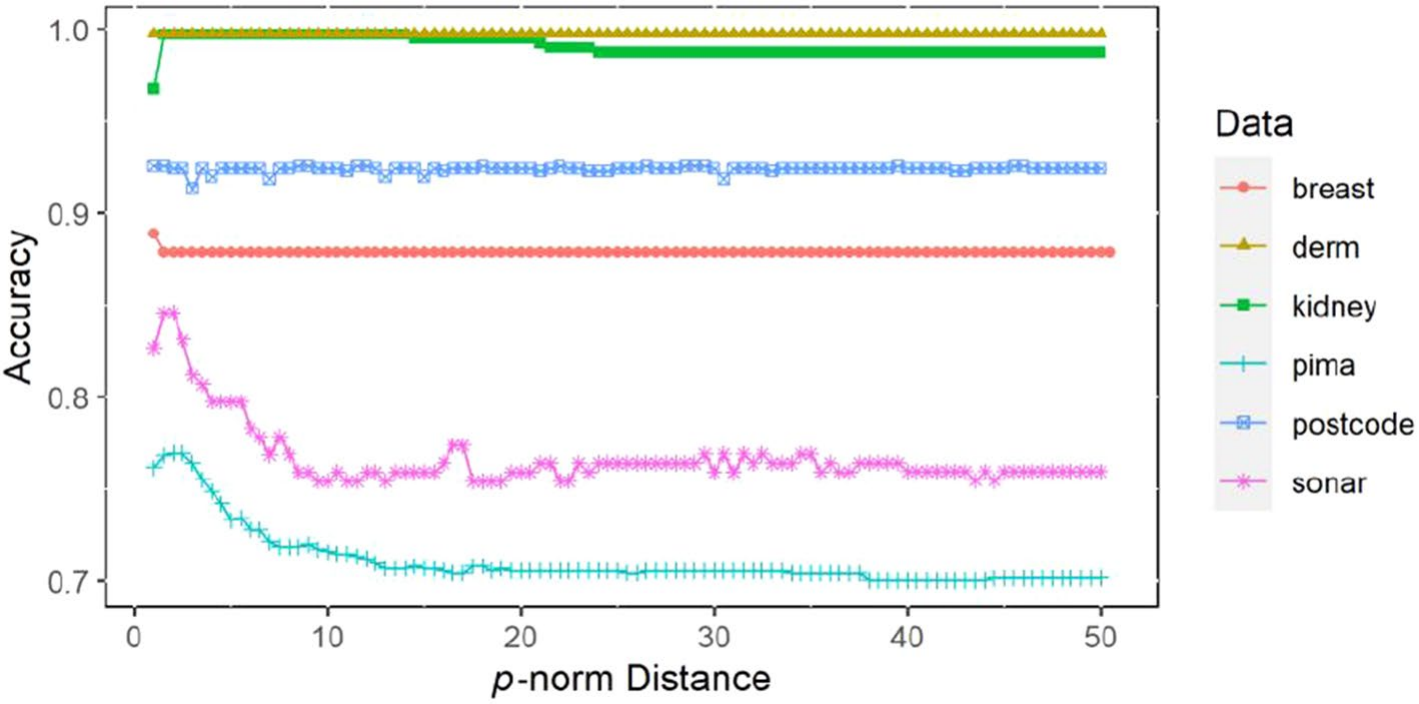
The fivefold cross-validation accuracy varies with *p*-norm distance based on 6 datasets.

**Figure 3:**
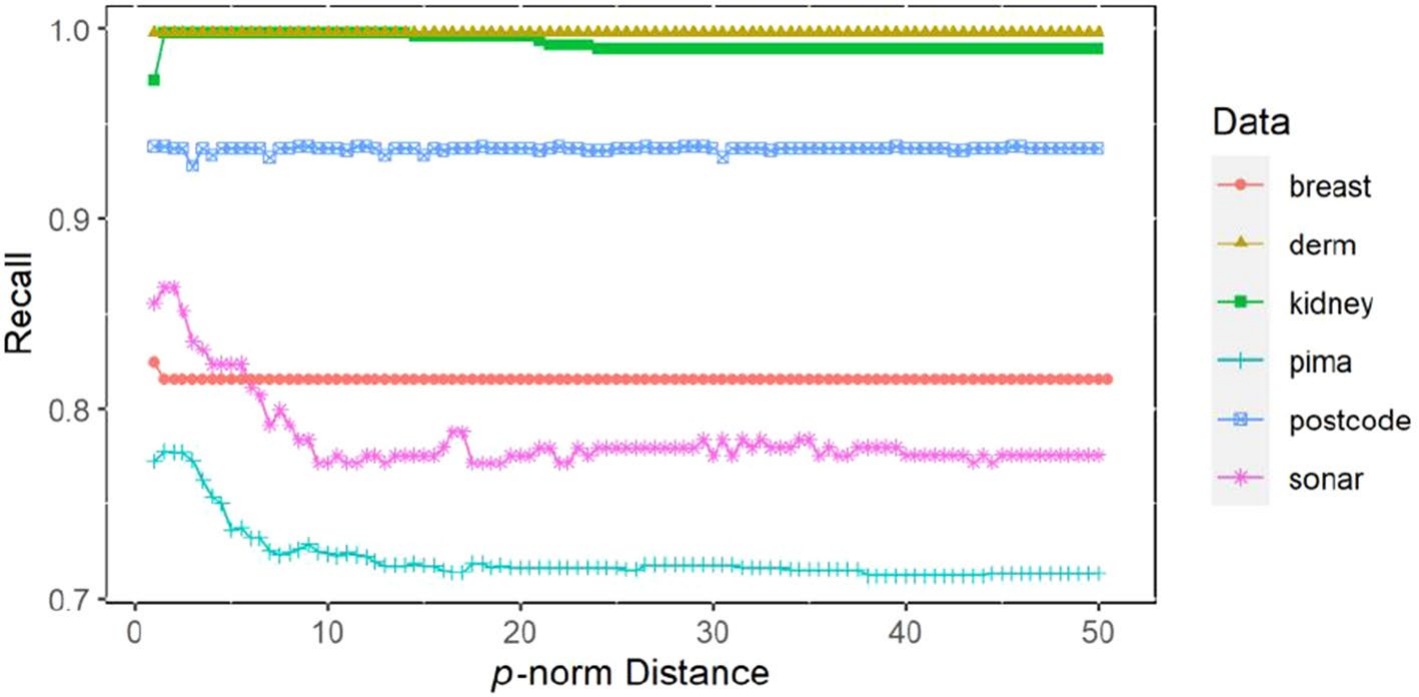
The fivefold cross-validation recall varies with *p*-norm distance based on 6 datasets.

**Figure 4:**
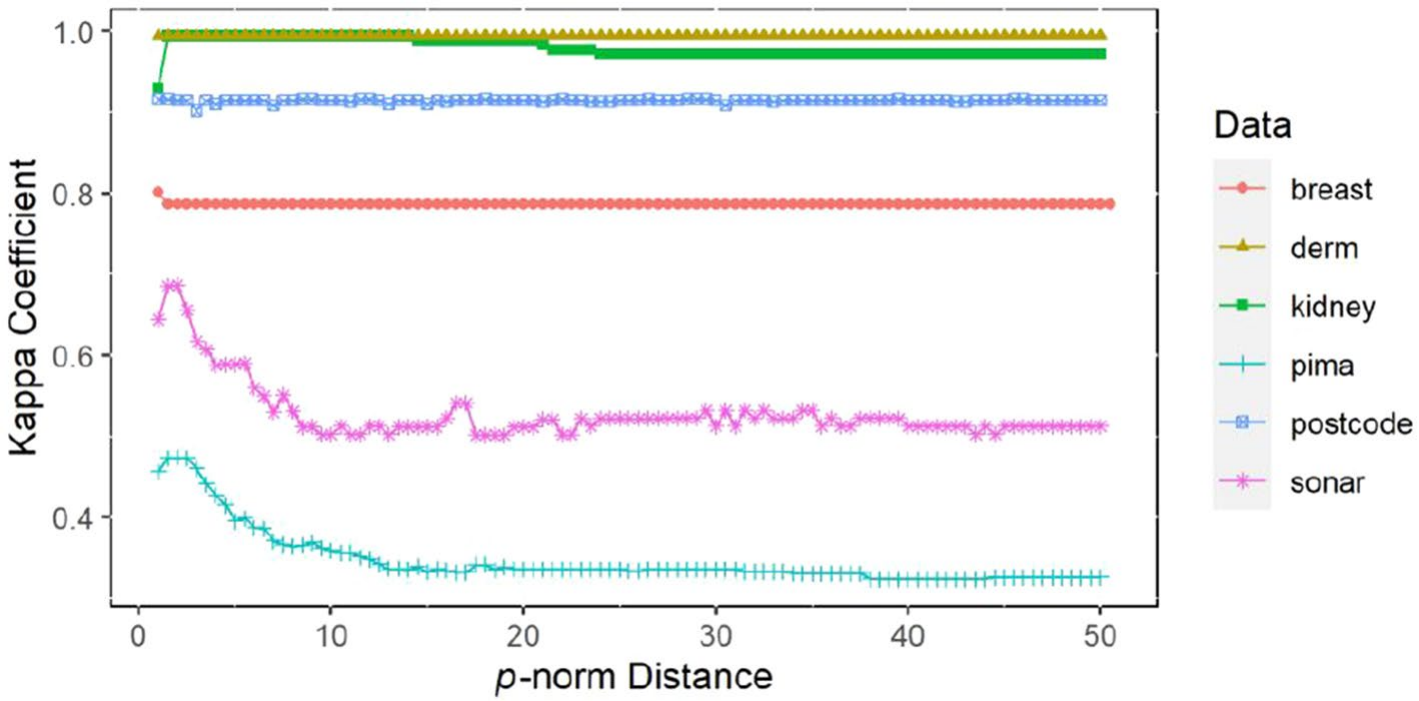
The fivefold cross-validation Kappa coefficient varies with *p*-norm distance based on 6 datasets.

For the W*p*Nt + SVM algorithm, different *p*-norm distances are set for different datasets because in the process of experimental analysis, it was found that different norms in the *p*-norm t kernel affect the classification performance of the SVM algorithm. For details, please refer to Figs. 2, 3 and 4, where $p \in [1,{\kern 1pt} {\kern 1pt} 50]$ and the step size is 0.5.

It can be clearly seen from Figs. 2, 3 and 4 that the accuracy, recall and Kappa coefficient using the W*p*Nt + SVM algorithm on the Sonar, Pima and Kidney datasets show significant changes with increasing *p*-norm distance. There is a gradual increase in these metrics until the highest point is reached, then an overall downwards trend with a slight fluctuation in the middle is observed. Finally, the results become stable. For the breast dataset, the accuracy, recall and Kappa coefficient of the model began to decline after reaching the highest point and basically remained at the same level. For the dermatology and postcode datasets, the accuracy, recall and Kappa coefficient of the model fluctuate only slightly with the change in the *p*-norm. From the visualized results in Figs. 2, 3 and 4, it can be concluded that setting different *p*-norm distances has a significant effect on the performance of the classification algorithm in some datasets, while the effect is relatively minimal in other datasets.

The above analysis verifies the influence of different *p*-norm distances on SVM classification performance through a set of cross-validation results, which often has strong randomness. We need to determine whether this significant or nonsignificant effect is necessary or random, so "statistical hypothesis testing" provides an important theoretical basis^45,46^. Next, a t test based on pairwise data is used to verify whether different *p*-norms have a significant impact on the classification performance of SVM algorithms in 6 datasets.

The specific operation steps are as follows.

(i) Given the two different norm distances $p_{1}$ and $p_{2}$, we perform 10 times fivefold cross-validation under the two norm distances. The two groups of classification evaluation indices of the SVM algorithm are obtained, including precision, recall and Kappa coefficient. They are represented as $x_{i}$ and $y_{i}$.
(ii) Let $$d_{i} = x_{i} - y_{i} \sim N(\mu ,\sigma^{2} )$$, $$H_{0} :\mu = 0;H_{1} :\mu \ne 0$$;
(iii) Let the statistic
  27 $$ t = \frac{{\overline{d}}}{{s_{d} /\sqrt n }}\sim t(n - 1), $$

where $\overline{d} = \frac{1}{n}\sum\nolimits_{i = 1}^{n} {d_{i} }$, $s_{d} = \sqrt {\frac{1}{n - 1}\sum\nolimits_{i = 1}^{n} {(d_{i} - \overline{d})^{2} } }$ and the significance level $\alpha = 0.05$;

(iv) The $t$ value in Eq. (27) is calculated. If $\left| t \right| > t_{1 - \alpha /2} (n - 1)$, then in a statistical sense, different *p*-norm distances have a significant effect on the SVM performance; otherwise, different *p*-norm distances do not have a significant effect on the SVM performance.

The null hypothesis and the alternative hypothesis in Step (ii) are equivalent to $H_{0} :$ the use of different *p*-norm distances has a significant effect on the classification performance of SVM, and $H_{1} :$ the use of different *p*-norm distances has no significant effect on the SVM classification performance. The critical value is $t_{0.975} (9) =$ 2.262 in Step (iv).

To compare whether different *p*-norm distances have significant effects on the performance of the proposed algorithm, the principle of the *p*-norm setting is as follows:

(i) Let $p \in [a,b]$, and the step size is $\lambda$;
(ii) The algorithm performance $MI_{i} ,i = 1,2,...s$ is calculated corresponding to different norms $p_{i}$;
(iii) When $\left| {MI_{i} - MI_{j} } \right| \ge \varepsilon ,1 \le i,j \le s$, the corresponding $p_{i}$ and $p_{j}$ are fixed.

For convenience, let $a = 1,b = 10,\lambda = 0.5,\varepsilon = 0.1$. If $\left| {MI_{i} - MI_{j} } \right| \ge \varepsilon$ does not exist in $[a,b]$, $\varepsilon$ is reduced appropriately. For the 6 datasets in the experiment, the 2-level *p*-norm distance is set, and the specific information is shown in Table 7.

**Table 7:** The *p*-norm distance setting in different datasets.

Dataset	*p*-norm distance
Kidney	$p_{1} =$ 1.5 $p_{2} =$ 2
Dermatology	$p_{1} =$ 1.5 $p_{2} =$ 2
Sonar	$p_{1} =$ 1.5 $p_{2} =$ 2
Pima	$p_{1} =$ 3 $p_{2} =$ 2
Postcode	$p_{1} =$ 1.5 $p_{2} =$ 3
Breast	$p_{1} =$ 2.5 $p_{2} =$ 3

According to the above steps, the test statistic is calculated and compared to the critical value. The test results are shown in Table 8.

**Table 8:** The statistical comparison test of the weighted t kernel SVM classification performance at the 2 level *p*-norm.

Dataset	Accuracy test			Recall test			Kappa coefficient test		
	$t$ Value	$\overline{x} - \overline{y}$	Sig	$t$ Value	$\overline{x} - \overline{y}$	Sig	$t$ Value	$\overline{x} - \overline{y}$	Sig
Kidney	6.488	0.0232	**Yes**	7.489	0.0279	**Yes**	4.421	0.0359	**Yes**
Dermatology	− 1.214	− 0.0009	No	− 0.9019	-0.0005	No	− 1.68	-0.0030	No
Sonar	3.074	0.0057	**Yes**	2.775	0.0054	**Yes**	2.874	0.0104	**Yes**
Pima	− 5.116	− 0.0060	**Yes**	− 3.283	-0.0055	**Yes**	− 5.254	− 0.0118	**Yes**
Postcode	1.937	0.0025	No	1.157	0.0015	No	1.934	0.028	No
Breast	1.5	0.0020	No	1.5	0.0017	No	1.496	0.0035	No

Sig: significance.

Significant values are in bold.

For the Kidney, Sonar and Pima datasets, the test results in Table 8 show that there is a significant difference in accuracy, recall and Kappa coefficient. For the other three datasets, there is no difference in accuracy, recall or Kappa coefficient at different *p*-norm levels, which is basically consistent with the results shown in Figs. 2, 3, and 4. In summary, it can be concluded that the change in the *p*-norm distance for different datasets will have different influences on the classification performance of SVM. In some datasets, such as the Sonar, Pima and Kidney datasets, the influence of the change in the *p*-norm distance is significant; in other datasets, such as the Postcode, Dermatology and Breast datasets, the influence is of the change in the *p*-norm distance is minimal. Therefore, when the kernel functions have the form of the *p*-norm distance, such as *p*-norm t kernel constructed in this paper and the traditional Gaussian kernel, we need to consider the influence of the norm distance on the performance of SVM and obtain the appropriate norm distance through experimental analysis to achieve the best classification prediction effect of SVM.

## Conclusions

For the classical SVM algorithm, the kernel function plays a crucial role in the classification prediction process because an appropriate kernel function can map samples to an appropriate feature space so that similar samples are close together and different samples are far apart. In view of this characteristic of the SVM algorithm, the *p*-norm distance t kernel is constructed according to the *t* probability density function, and a strict theoretical proof is given. To make use of the advantages of different types of kernel functions, the kernel functions are combined. The affinity matrix is redefined according to the local kernel polarization, and then an optimization model is established to solve the weight coefficients and kernel parameters. The weighted *p*-norm t kernel is applied to the SVM classification. Experimental analysis on six datasets shows that the proposed weighted *p*-norm t kernel can effectively improve the classification prediction performance of the SVM algorithm compared with the traditional single kernel function. Finally, the influence of the *p*-norm distance on the performance of the SVM algorithm is analysed based on a statistical comparison test. It is concluded that for different datasets, different norm distances will have different effects on the performance of the algorithm, some of which are significant and some of which are minimal.

The multiple kernel method based on improved local polarization in this paper is applied to SVM classification. Our method is also suitable for dimensionality reduction, kernel clustering and medical drug screening. In future work, this method will be improved and generalized in these research directions. However, the proposed method in this paper is only a simple linear combination of multiple kernel functions. There is no complete and effective theoretical basis for the selection and combination of kernel functions; the optimization of kernel weights and kernel parameters still faces the problem of nonconvergence, which needs to be further solved.

## Supplementary Information

Supplementary Information 1.

Supplementary Information 2.
