# Supplementary figures and images for: Weighted p-norm distance t kernel SVM classification algorithm based on improved polarization
Source: Sci Rep. 2022 Apr 13;12:6197. doi: 10.1038/s41598-022-09766-w (PMC9008017; doi:10.1038/s41598-022-09766-w)

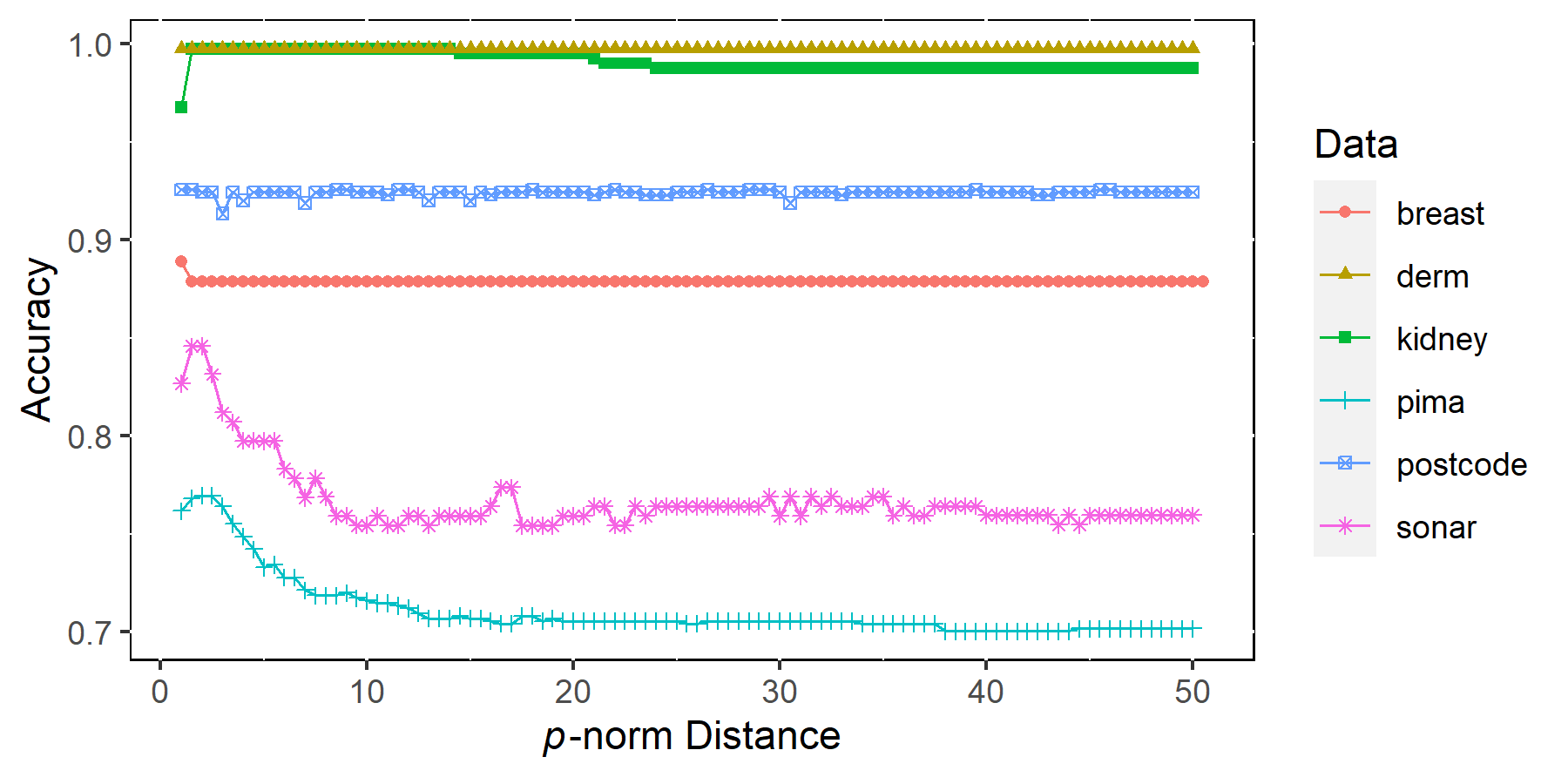

Supplement: Supplementary file 1 — Supplementary Information 1. [file 41598_2022_9766_MOESM1_ESM.zip › Code/paper plot/p-norm acc result.tiff]

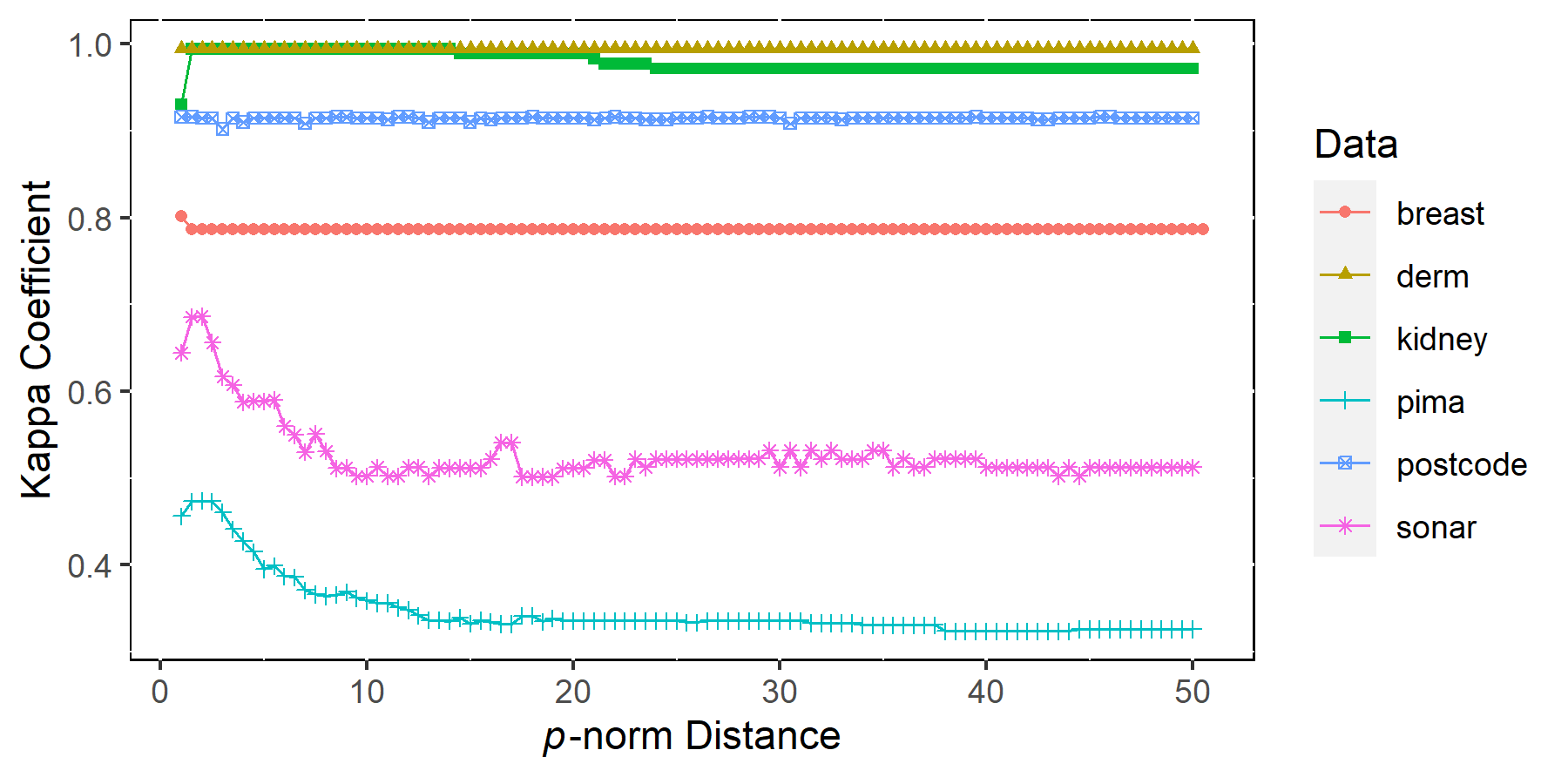

Supplement: Supplementary file 1 — Supplementary Information 1. [file 41598_2022_9766_MOESM1_ESM.zip › Code/paper plot/p-norm kappa result.tiff]

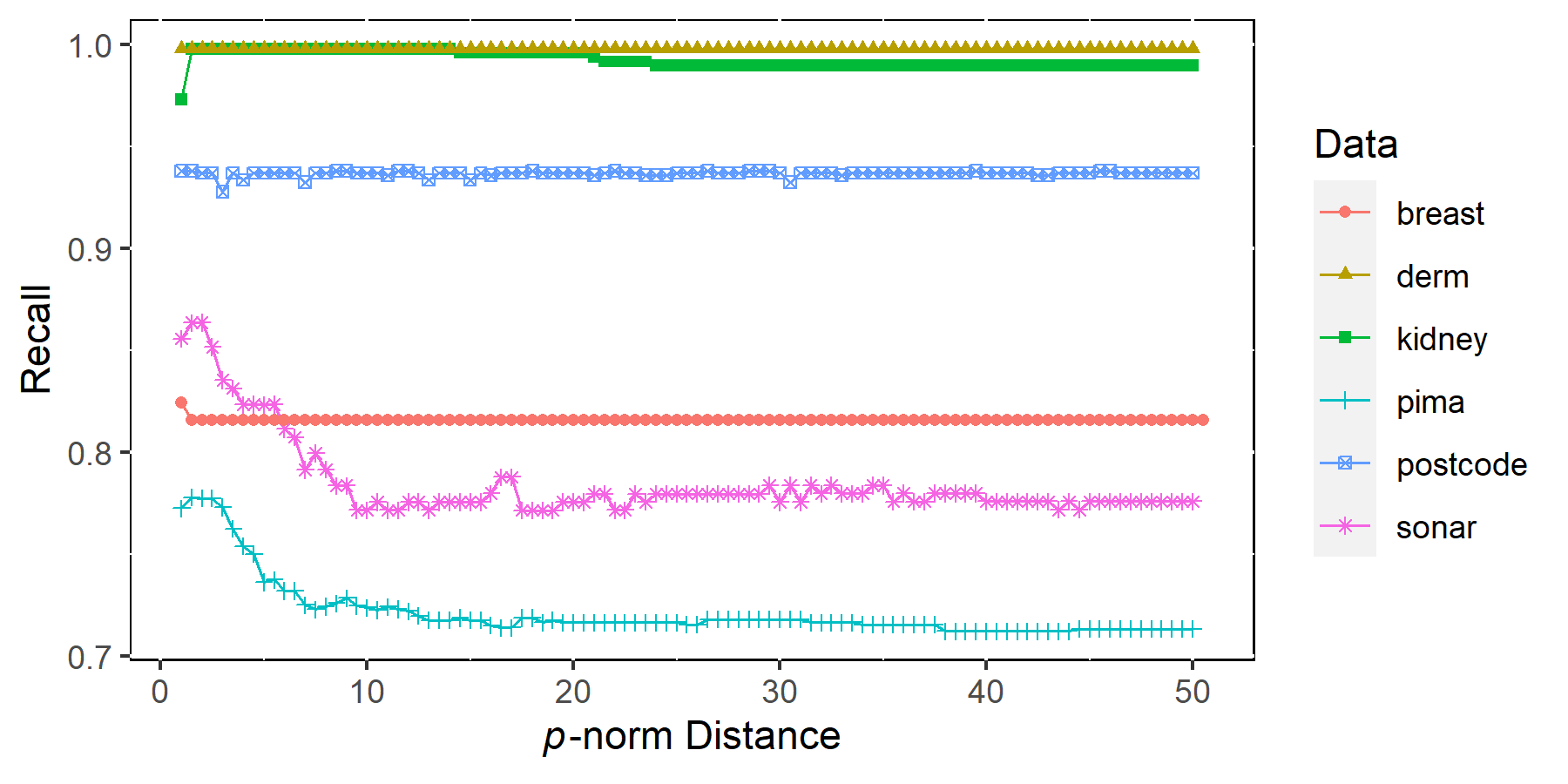

Supplement: Supplementary file 1 — Supplementary Information 1. [file 41598_2022_9766_MOESM1_ESM.zip › Code/paper plot/p-norm recall result.tiff]

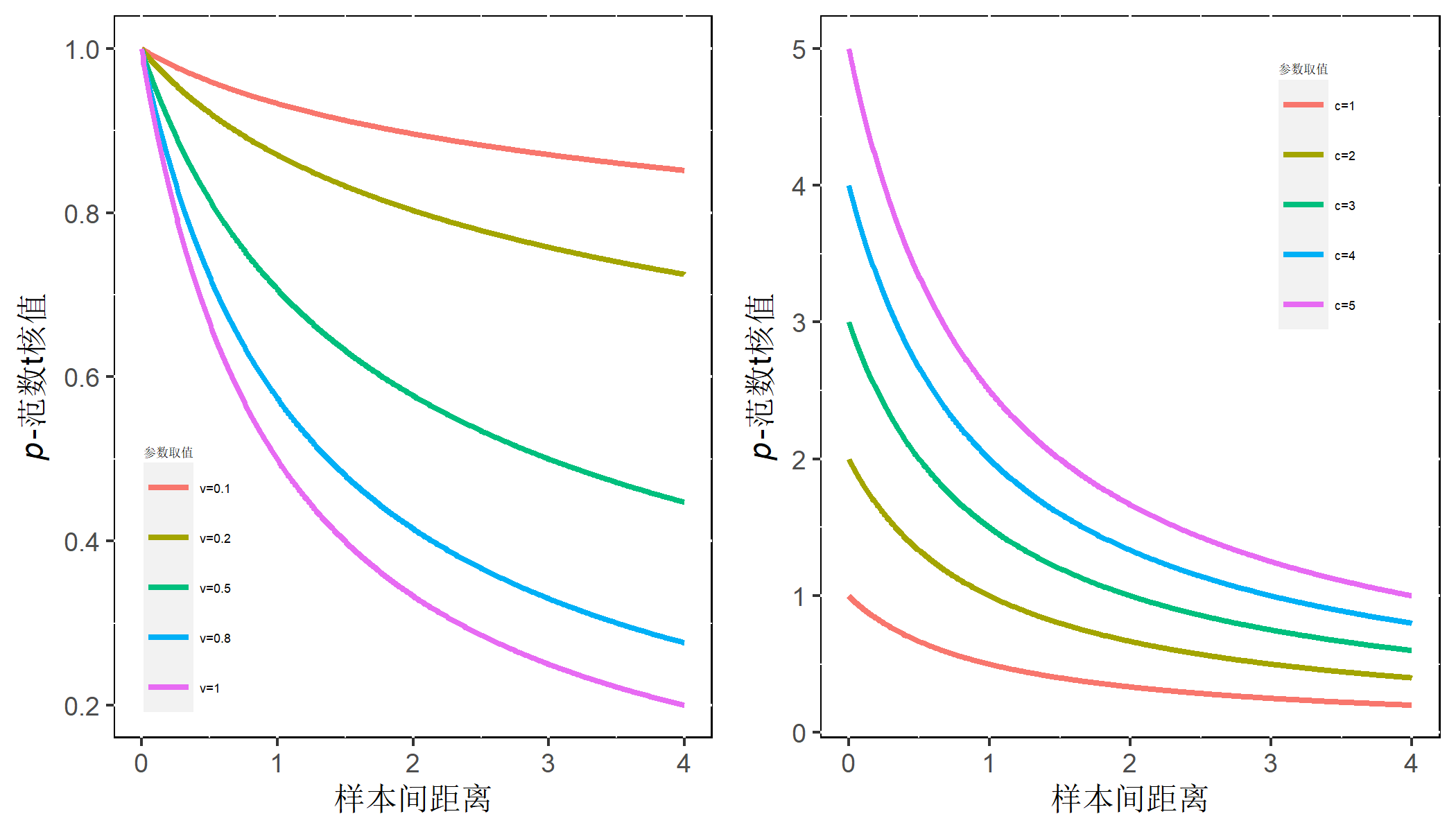

Supplement: Supplementary file 1 — Supplementary Information 1. [file 41598_2022_9766_MOESM1_ESM.zip › Code/paper plot/ptkernel.tiff]
